# Single-Plant Selection at Ultra-Low Density Enhances Buffering Capacity of Barley Varieties and Landraces to Unpredictable Environments and Improves Their Agronomic Performance

**DOI:** 10.3389/fpls.2022.838536

**Published:** 2022-02-18

**Authors:** Athanasios L. Tsivelikas, Hajer Ben Ghanem, Adil El-Baouchi, Zakaria Kehel

**Affiliations:** ^1^Genetic Resources Section, International Center for Agricultural Research in the Dry Areas (ICARDA), Rabat, Morocco; ^2^Field Crop Laboratory, National Institute of Agricultural Research, University of Carthage, Tunis, Tunisia; ^3^African Integrated Plant and Soil Research Group (AiPlaS), AgroBioSciences, Mohammed VI Polytechnic University, Ben Guerir, Morocco

**Keywords:** barley, buffering capacity, drought conditions, single-plant selection, ultra-low density, yield compensation

## Abstract

Rainfall and temperature are unpredictable factors in Mediterranean environments that result in irregular environmental conditions for crop growth, thus being a critical source of uncertainty for farmers. This study applied divergent single-plant selection for high and low yield within five barley varieties and two Tunisian landraces under semi-arid conditions at an ultra-low density of 1.2 plants/m^2^ for two consecutive years. Progeny evaluation under dense stands following farmers’ practices was conducted in two semi-arid locations in Tunisia during one cropping season and in one location during a second season, totalling three environments. The results revealed significant genotypic effects for all recorded agronomic and physiological traits. No genotype × environment interaction was shown for biological yield, implying a biomass buffering capacity for selected lines under different environmental conditions. However, genotype × environment interaction was present in terms of grain yield since plasticity for biomass production under drought stress conditions was not translated directly to yield compensation for some of the lines. Nevertheless, several lines selected for high yield were identified to surpass their source material and best checks in each environment, while one line (IH4-4) outperformed consistently by 62.99% on average, in terms of grain yield, the best check across all environments. In addition, improved agronomic performance under drought conditions induced an indirect effect on some grain quality traits. Most of the lines selected for high yield maintained or even improved their grain protein content in comparison to their source material (average increase by 2.33%). On the other hand, most of the lines selected for low yield indicated a poor agronomic performance, further confirming the coherence between selection under ultra-low density and performance under dense stand.

## Introduction

On a global scale, barley (*Hordeum vulgare* L. subsp. *vulgare*) ranks fourth among cereals in terms of production quantity, after wheat, maize, and rice, providing nutrient benefits for both livestock and humans (Newton et al., 2011; FAOSTAT, 2021). Barley is a member of the grass family (Poaceae). It is a versatile crop with the ability to adapt to unfavourable conditions that distinguish it as one of the best models and most suited crops for studying adaptation to climate change (Dawson et al., 2015). Despite its resilient nature to climate disruptions, high relative yield gap rates have been estimated for barley crop, ranging between 12 and 75% for the rainfed systems in Europe (Schils et al., 2018) and up to 25% for the rainfed barley fields in Alberta, Canada (Chapagain and Good, 2015). Volatile climate conditions, management practices, genetic factors, as well as social restrictions on the use of inputs and economic disincentives to intensify crop production are amongst the main causes of considerable variation and stagnated or even declined yields for barley and other major crops (Peltonen-Sainio et al., 2009; Lin and Huybers, 2012; Mueller et al., 2012; Tokatlidis, 2014; Ray et al., 2015; Hochman and Horan, 2018).

In this regard, climate change seems to induce severe yield losses for barley crops mainly due to an increase in maximum temperature during the grain filling period causing heat stress, as well as due to an increased frequency of drought events during the stem elongation period (Brisson et al., 2010; Bento et al., 2021). Climate change impacts and, hence, their consequences do not follow an evenly distributed spatial pattern with their magnitude varying from region to region (Trenberth, 2011). Among the most fragile areas, the Mediterranean region has been well recognised as a prominent climate change hot spot (Diffenbaugh and Giorgi, 2012; Alessandri et al., 2014). Mediterranean environments are characterised by high inter-annual variability of temperature and rainfall patterns, increasing the uncertainty of maintaining production at higher levels (Cammarano et al., 2019). This unforeseen variation is likely to affect yield and yield quality directly, due to impact on crop physiology and indirectly, due to alterations in nutrient mineralisation and availability for crops (Henson, 2011; Cammarano et al., 2019).

However, the resource-limited regime in crop stands results in plant-to-plant competition due to the concurrent demand of individual plants for the available resources (Weiner and Damgaard, 2006). As highlighted by Farrior et al. (2013), resource limitation creates incentives for plants to over-invest in resource capture at levels that are suboptimal for the productivity of a plant in isolation but pay off for the plant interference with the others, such as investment in height growth for light capture or in fine roots for belowground resources. Competition between individuals within a crop may lead to developmental dissimilarities and intra-crop inequality (Weiner and Damgaard, 2006; Tokatlidis, 2017). This intra-crop inequality, in turn, further aggravates the unequal share of limited resources, thus intensifying inter-plant competition, functioning as a vicious circle between plant asymmetry and competition that perpetuates all along the crop cycle. Evidently, this condition affects plant growth detrimentally to crop yield performance (Pan et al., 2003; Pagano and Maddonni, 2007). Complexity is exacerbated under high stand densities since plants are more prone to early established inequalities leading to pronounced morphological and physiological differences which in turn affect resource use efficiency during critical developmental stages of the crop (Tollenaar et al., 2006; Rossini et al., 2011; Yan et al., 2017; Sher et al., 2018).

Planting density is one of the key factors in achieving crop uniformity by minimising interplant competition and ensuring an equal share of resources, eventually attaining maximum profitability. Recommending an optimum planting density is not an easy task, since the relationship between planting density and grain yield is governed by several parameters that fall under the genotype, environment, and crop management effect (Assefa et al., 2016, 2018; Carciochi et al., 2019; Bastos et al., 2020). In wheat, for example, Bastos et al. (2020) concluded that for high yielding environments and less limited resources the number of plants required to maximise yields was very low and below any commercially recommended number of plants for this crop, while for low yielding environments a higher density was needed to sustain maximum yields. Likewise, according to Matsuyama and Ookawa (2020), a lower seeding rate than the one commonly practiced in Japan was more suitable in achieving high yields and improved lodging resistance for those wheat cultivars that were characterised by a high number of grains per spike when these cultivars were planted in soils with abundant resources. In barley, most of the research concludes that a seeding rate which establishes between 300 and 360 plants m^–2^ is usually the optimal one (Thomason et al., 2009; O’Donovan et al., 2012; Perrott et al., 2018). However, the recommended density can vary considerably depending on the field properties and climate conditions or even on the interaction with the genotype (Jedel and Helm, 1995; O’Donovan et al., 2012; Bekele et al., 2020). Undoubtedly, the main constraint to define optimum plant density lies on the large environmental variability that occurs in a particular zone across seasons, as well as on the unpredictability of the inter-annual variation in terms of weather conditions, and predominantly in the amount and distribution of atmospheric precipitations in the rain-fed cropping systems (Tokatlidis, 2014). Therefore, Tokatlidis (2017), highlights the importance of breeding to target varieties that are characterised by homeostasis, that is the ability to withstand external forces that induce acquired plant-to-plant inequality and concomitant intra-crop competition, as well as by density-independence, to perform satisfactorily at relatively low densities.

Since intra-crop inequality and inter-plant competition are related to high densities, a condition for selection under ultra-low density that excludes plant-to-plant interference for resources (i.e., nil-competition) is a prerequisite. Such a condition exploits the honeycomb breeding model (Fasoulas, 1988, 1993). Owing to their systematic entry arrangement, locating each plant in the centre of a circular replicate/ring to ensure increased local control and allocating the plants of each entry in a moving triangular grid spread across the whole field for an effective sampling of soil heterogeneity, honeycomb designs objectively evaluate sister-lines and apply single-plant selection under a pattern of ultra-low planting density (Fasoulas and Fasoula, 1995). The nil-competition regime maximises the phenotypic expression of genetic differences among individuals, facilitating, further, the detection of desirable genotypes (Kyriakou and Fasoulas, 1985; Fasoula and Fasoula, 2002; Tokatlidis et al., 2010). Moreover, the selection under ultra-low density erases the confounding effects of competition on the identification of high yielding genotypes, induced by the negative relationship between yielding and competitive ability (Kyriakou and Fasoulas, 1985; Chatzoglou and Tokatlidis, 2012; Ninou et al., 2014), while attaining greater heritability by minimising the acquired variance arising from non-genetic sources (Fasoula and Fasoula, 2002; Tokatlidis, 2015). The computation of mathematical parameters that account for relative plant yield efficiency and stability of performance is easily performed and can be applied from the early stages of selection for selecting superior plants, thus reducing the time frame required for the release of improved varieties.

Considering the challenges imposed by climate variation and volatility and the need to expand the range of optimum planting density in field crops, the development of barley cultivars with an innate buffering capacity to perform well enough under varying and unpredictable climate conditions and making optimum use of the available resources, sound as a prudent approach to reduce the gap between actual and attainable yield in barley crop. Hence, the objective of the present study was to investigate the performance and buffering capacity of barley lines under favourable and drought stress conditions in Tunisia. These lines were derived from three commercially released cultivars and two Tunisian landraces, using single-plant selection at ultra-low density. Furthermore, the potential to exploit latent or *de novo* variation within barley cultivars for the development of high-yielding lines with elevated homeostasis and competent qualitative traits is discussed.

## Materials and Methods

### Plant Material

To obtain the barley lines evaluated in this study, selection started in 2014 cropping season among five commercially released cultivars in Tunisia (Imen, Kounouz, Lemsi, Manel, and Rihane) and two Tunisian landraces (Ardhaoui and Djebali) planted under the ultra-low density of 1.2 plants/m^2^ according to an R-7 honeycomb field layout (Fasoulas and Fasoula, 1995). The selection between entries was based on the computation for each of the entries of the three parameters described by Fasoula and Fasoula (2000), that is (i) the entry’s mean ($\bar{x}$), (ii) the entry’s standardised mean $\left(\bar{x}/s\right)$, and (iii) the entry’s standardised selection differential $\left(\frac{{\bar{x}}_{sel}-\bar{x}}{s}\right)$. Then, divergent single-plant selection for high and low yield within the top entries was applied by the moving-circle procedure (Fasoulas and Fasoula, 1995) to form the first cycle selected lines. These lines along with the best commercial checks of the region were further subjected to selection in the following cropping season, by applying the same principles of single-plant selection for high and low yield under an ultra-low-density regime of 1.2 plants/m^2^ according to an R-21 honeycomb field layout (Fasoulas and Fasoula, 1995). In both years, the selected high-yielding plants were the ones that showed the highest grain weight compared with the mean of the 36 surrounding plants (i.e., 0.027 selection pressure). Low-yielding individuals were identified using the same selection pressure, but in this case, selected plants should weigh at least 10 g of grains and then bulked according to the source material, to get enough seeds for the next selection cycle and for further evaluation. The whole procedure resulted in 12 first cycle lines (8 high yielding and 4 low yielding) and 38 second cycle lines (30 high yielding and 8 low yielding) to be assessed in the next seasons’ dense stand trials. The honeycomb experimental field layouts and the selection procedure applied for two consecutive years are described in detail by Ben Ghanem et al. (2018). A summary of the selection history of the progeny lines is given in Table 1.

**TABLE 1:** Selection history of the single-plant progeny lines derived through divergent selection at ultra-low density and evaluated at the dense stand trials (modified by Ben Ghanem et al., 2018).

Source material	First cycle HY lines	First cycle LY lines	Second cycle HY lines	Second cycle LY lines
Ardhaoui	AH9, AH10	AL0	AH9-H1, AH9-H2, AH9-H3, AH10-H1, AH10-H2, AH10-H3	AH9-L0, AH10-L0
Imen	IH4, IH16, IH17	IL0	IH4-H1, IH4-H2, IH4-H3, IH4-H4, IH16-H1, IH16-H2, IH16-H3, IH17-H1, IH17-H2, IH17-H3, IH5-VS	IH4-L0, IH16-L0, IH17-L0
Djebali	DH2, DH12	DL0	DH2-H1, DH2-H2, DH2-H3, DH2-H4, DH2-H5, DH12-H1, DH12-H2, DH12-H3, DH14-VS	DH2-L0, DH12-L0
Manel	MH18	ML0	MH18-H1, MH18-H2, MH18-H3	MH18-L0
Rihane	–	–	RH8-VS	–

*The coding of lines is based on two letters and the number of the selected plant. In the case of the bulk sample, this is indicated with 0. The first letter indicates the source material from which the line has been selected (A stands for Ardhaoui, I for Imen, D for Djebali, M for Manel, and R for Rihane). The second letter indicates whether the selection is based on high yield (H) or low yield (L). Cases indicated with VS, stand for visual selection.*

### Field Evaluation Trials

In the 2016 growing season, the 50 first and second cycle selected lines along with five checks (source seed lots of Imen, Ardhaoui, Djebali, Manel, and Rihane) were planted as dense stand field trials at the National Agricultural Research Institute of Tunisia (INRAT) experimental stations in El Kef (36° 14′ N; 8^°^ 27′ E; 518 m) and Mornag (36^°^ 37′ N; 10^°^ 17′ E; 54 m) in Tunisia. These materials were also planted as dense stand trial the following growing season at Mornag experimental station. The two research stations represent two distinct production environments for Tunisia. Mornag is characterised by clay soil and average annual precipitation of 450 mm. El Kef is characterised by clay loam soil and average annual precipitation of 452 mm with barley being the most common rainfed crop of the region. The monthly precipitation at the two experimental sites for the growing seasons during which the selection and evaluation trials were held is given in Table 2.

**TABLE 2:** Monthly precipitation at the two experimental sites for the growing seasons of selection and evaluation trials.

Site	Growing season	Trial type	September	October	November	December	January	February	March	April	May	June	July	August	Total
El Kef	13/14	Selection	52	25	103	52	52	35	81	8	53	17	0	1	479
El Kef	14/15	Selection	20	35	43	74	70	66	66	0	22	2	5	46	449
El Kef	15/16	Evaluation	18	26	48	5	55	13	89	30	32	5	0	2	325
Mornag	15/16	Evaluation	6	57	44	23	18	40	63	14	25	0	0	5	295
Mornag	16/17	Evaluation	80	33	77	140	53	36	4	16	0	19	0	0	458

A non-replicated augmented design field trial was established in all cases, with five incomplete blocks and 15 entries per block. Plots were composed of four rows of 2.5 m long, each with 0.25 m spacing between rows, occupying a plot area of 2.5 m^2^. Plot by plot distance within the same alleyway was 0.75 m and between alleyways 1.5 m. All trials were planted under a uniform seed rate of 360 seeds/m^2^. To ensure the robust establishment of field plots, seeds were treated before planting with Celest top [Diféconazole (25 g/L) + Fludioxonil (25 g/L) + Thiamethoxam (262.5 g/L)] at a rate of 200 ml/hl of seeds. Basic fertiliser in the form of diammonium phosphate (18-46-0) was applied before planting at a rate of 100 kg/ha. Complete weed control was attained by chemical applications (Axial: pinoxaden (100 g/L) + cloquintocet-methyl (25 g/L) at a dose of 1 L/ha for the narrow leaf weeds and Zoom: dicamba (66%) + Triasulfuron (4%) at a dose of 180 g/ha for the broadleaf weeds) and hand weeding. Two spring foliar spray applications of Ogam [Kresoxim-methyl (125 g/L) + Epoxiconazole (125 g/L)] at a rate of 0.7 L/ha were applied as a preventive measure to minimise yield reductions due to fungal diseases. The harvest took place beginning of June, and all four rows per plot were harvested.

### Data Records for Agronomic and Physiological Traits

Several agronomic and physiological traits were recorded across the three environments. Regarding agronomic traits, biological yield (BY: t/ha) and grain yield (GY: t/ha) per plot were measured at maturity and, harvest index (HI) was derived as the quotient between grain and biological yield. Plant height (PH) was measured at maturity from five randomly selected plants within each plot and recorded as the distance in centimetres from soil level to the tip of spikes excluding the awns. Spike length (SL) was recorded as the average of ten representative spikes of each plot from the base up to the tip of the spike. Each of these spikes was then threshed individually and the average grain weight per spike (SGW) expressed in g for each of the entries was also recorded. Powdery mildew (PM) reaction was scored based on the prevalence of the disease at the seedling stage at El Kef and Mornag stations during the 2016 cropping season based on a disease severity scale from 1 to 5, with 1 as no symptoms and 5 as highly susceptible.

For physiological parameters, measurements were performed only in the 2016 growing season in the two locations where the trials had been planted. Soil Plant Analysis Development (SPAD) values at the heading stage SPAD were measured on fully expanded flag leaves of three representative plants of each plot using a MINOLTA SPAD 502 Plus chlorophyll meter. Leaf canopy temperature (LCT) was recorded as the average of five representative positions within each plot using an infrared scantemp 440 thermometer. Chlorophyll fluorescence F_0_, F_m_, and F_v_ parameters were measured at heading time at the fully expanded flag leaves of the three representative plants within each plot, for which the SPAD values were also taken, using an OPTI-SCIENCE 0530 + handheld portable fluorometer. These measurements were then used to calculate the ratios F_v_/F_m_ and F_v_/F_o_ and thus, test for differences in the activity of photosystem II (PSII).

### Grain Quality Parameters

Representative grain samples from all field plots of the two locations planted in the 2016 cropping season were transferred and evaluated in International Center for Agricultural Research in the Dry Areas (ICARDA) Quality Laboratory. In particular, grain colour, morphology, physiochemical parameters, and β-Glucans content were assessed.

#### Grain Morphology and Grain Colour

Random samples of 70 grains were received from all seed lots representing each plot at the field and scanned using a flatbed scanner (CanoScan LiDE 220; Canon). The images collected were analysed using Grainscan software (Whan et al., 2014), which generated the morphological and colour profile for every single grain. Grain morphology traits, such as perimeter in mm (PRM), grain length in mm (LNG), and width in mm (WDT) were calculated for each sample as means of the 70 seeds. In addition, a colour channel intensity output similar to the standardised CIELAB colour space produced by the software (Whan et al., 2014). The GrainScan colours (ColCha1, ColCha2, and ColCha3) were therefore considered proxies for L, a, and b, respectively, representing the lightness of the colour, green or magenta, and blue or yellow.

#### Physiochemical Parameters

Barley protein content (PRT) and starch (STRCH) were determined using near-infrared spectroscopy (NIR, Infratec 1241, Foss). To determine the β-glucan content (β-GLC) the calcofluor-fluorimetric method using a flow analyser (SKALAR san^++^) was employed. Before this determination, an acid extraction was carried out according to the method recommended by the European Brewery Convention (Manzanares and Sendra, 1996). Briefly, 100 mg of barley flour was weighed. A volume of 10 mL distilled water was added jointly with 100 μL of alpha-amylase and dispersed with a vortex mixer. Then, the tube was boiled for 1 h and after cooling, 10 ml of sulphuric acid was added. The mixture was homogenised, boiled for 10 min, cooled to room temperature, and finally centrifuged and the aliquot filtered prior to being loaded into the sampler of the flow analyser.

### Data Analysis

Raw data values for agronomic, physiological, and grain quality traits were analysed by employing the analysis of variance using linear mixed models. For this purpose, locations and years were combined into a single factor (environment). Genotypes (entries), environments, and entry × environment interaction were considered as fixed effects, while the block effect and the plot effect nested in each block as random. Based on this model, the best linear unbiased estimations (BLUEs) were computed for all recorded traits. To identify the best performing lines across and within each environment, entries were analysed in relation to their source material by performing a GGE biplot analysis based on the grain yield BLUEs values of the entries in each distinct environment. In addition, Pearson correlation coefficients between all recorded traits were computed and a heat map was generated based on correlations. Statistical analysis was performed with JMP statistical package ver. 14.0.0.

## Results

### Agronomic Performance Traits

The combined ANOVA revealed a significant effect of the environment for BY, GY, PH, and SGW traits, while there was no effect for HI, SL, TKW, and PM (Table 3). The three environments differed considerably in terms of annual precipitation with Mornag_16 being the driest one with 295 mm of rainfall, followed by El Kef_16 receiving 325 mm of rain. A very different annual precipitation pattern was observed the following year when the Mornag_17 environment recorded a total of 458 mm of rain (Table 2). Hence, in terms of BY the lowest values were recorded at the driest environment Mornag_16 with a mean value of 3.75 t/ha reduced by 46 and 48% compared to the respective BY values in El Kef_16 and Mornag_17 (Table 4). The same trend was also revealed for GY with the driest environment Mornag_16 to indicate a mean value of 1.46 t/ha, being significantly lower from the mean GY in El Kef_16 and Mornag_17 with the difference exceeding 1 t/ha (Table 4). Regarding the PH, distinct values were recorded among the three environments, with Mornag_17 demonstrating the tallest stands with an average value of 81.63 cm, followed by a 13 and 33% reduction at El Kef_16 and Mornag_16 environments, respectively (Table 4). Furthermore, the driest environment Mornag_16 revealed the lowest values for the SGW, with the mean value of 2.13 g being by 7% reduced by the respective value in the El Kef_16 environment (Table 4).

**TABLE 3:** Genotypic and environmental effects and their interaction on the agronomic traits of barley lines selected under ultra-low density when evaluated under dense stand trials in different environments in Tunisia.

A. Traits recorded in three environments					
**Source of variation**	**DF**	**BY**	**GY**	**HI**	**PH**
Entry	54	36.9***	46.2***	36.3***	52.6***
Environment	2	4.8***	36.5***	3.3	5.6***
Entry × Environment	108	9.4	12.5*	12.2**	12.0

**B. Traits recorded in two environments**					

**Source of variation**	**DF**	**SL**	**SGW**	**TKW**	**PM**
Entry	54	12.5***	35.4***	15.9***	32.5***
Environment	1	2.1	35.4**	31.9	3.6
Entry × Environment	54	17.9	35.4*	31.5	33.6

*Significant at α = 0.05; **Significant at α = 0.01; ***Significant at α = 0.001.

**TABLE 4:** Agronomic traits means and confidence intervals of barley entries evaluated in different environments in Tunisia.

	El Kef_16			Mornag_16			Mornag_17		
Trait	Mean	Lower 95%	Upper 95%	Mean	Lower 95%	Upper 95%	Mean	Lower 95%	Upper 95%
BY (t/ha)	6.90	6.522	7.276	3.75	3.495	4.007	7.18	6.672	7.697
GY (t/ha)	2.49	2.275	2.713	1.46	1.325	1.596	2.78	2.516	3.039
HI	0.36	0.339	0.384	0.38	0.3567	0.400	0.37	0.358	0.392
PH (cm)	71.04	68.717	73.362	54.73	53.209	56.258	81.63	79.749	83.505
SL (cm)	7.23	7.059	7.398	7.09	6.950	7.228	NA	NA	NA
SGW (g)	2.29	2.218	2.366	2.13	2.037	2.216	NA	NA	NA
TKW (g)	34.63	32.767	36.484	33.53	31.195	35.868	NA	NA	NA
PM	2.67	2.545	2.788	3.19	3.037	3.337	NA	NA	NA

*NA, Not applicable, measurements not made.*

**FIGURE 1:**
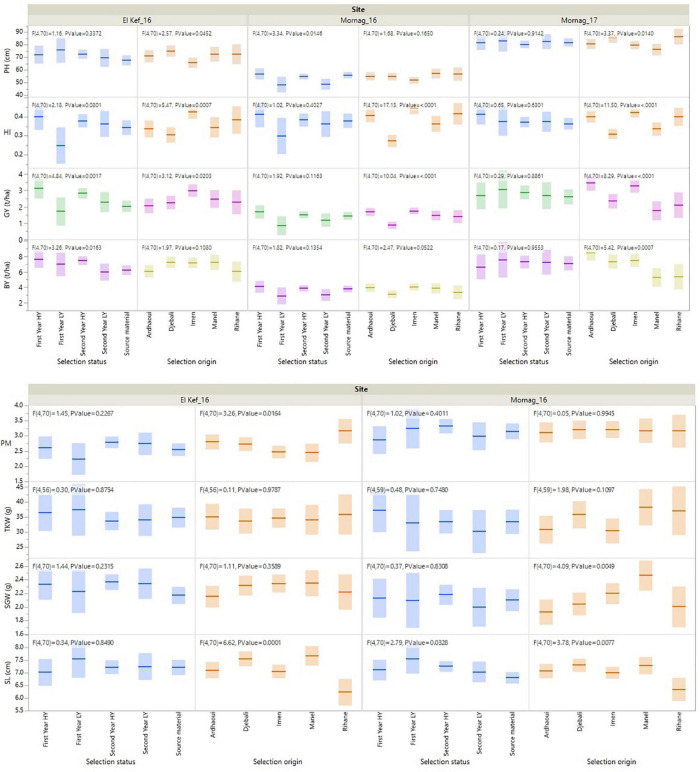
Fit of means for the agronomic traits of barley lines selected under ultra-low density when evaluated under dense stand trials in distinct environments in Tunisia.

Significant entry effects were revealed for all the recorded agronomic traits (Table 3). Almost for all traits the effect of selection status, i.e., first- and second-year HY and LY lines and source materials, as well as the effect of the source variety/landrace of the derived lines was significant (Figure 1). More specifically, the first- and second-year HY lines recorded the highest BY values at El Kef_16 with a total biomass of 7.65 and 7.46 t/ha, respectively, surpassing the original genotypes by 20%. Moreover, for the same environment, the first- and second-year HY lines showed the highest GY with 3.14 and 2.84 t/ha outperforming on average the source materials by 36 and 23%, respectively (Figure 1). No significant effects were found for the HI based on the selection status of the lines, even though a clear trend for high HI values was revealed for the first year HY lines that recorded a mean HI value of 0.41 across the three environments compared to the 0.36 HI value of the source materials. Concerning PH, the first- and second-year LY lines showed the lowest values at the Mornag_16 environment, with significantly shorter stands by a minimum of 6 cm compared to all other lines (Figure 1). For the agronomic traits recorded in two environments, the first-year LY lines revealed the longest spikes in the Mornag_16 environment with an average of 7.56 cm, longer by 11% in comparison to the average length of the source materials. The same trend for the first year LY lines was revealed also at the Kef_16 environment, however, in this case, the differences did not reach the significance level (Figure 1). Despite the differences in terms of SL, no significant effects were found for the SGW based on the selection status of the lines in both environments. The same was also true for the TKW and PM, traits for which the selection status of the lines did not reveal any significant difference (Figure 1).

When the effect of the source variety/landrace of the derived lines was assessed, the lines derived from variety Imen appeared consistent high values across the three environments with a mean BY of 6.26 t/ha producing on average 12 and 21% more biomass than the lines acquired from Manel and Rihane varieties, respectively (Figure 1). For the lines originated from the two landraces, the ones from Ardhaoui produced high biomass with a mean BY value of 6.20 t/ha across the three environments, while the lines from Djebali showed contrasting results being among the high biomass producing lines for the favourable environment of Mornag_17 but ranked amongst the least producing lines for the dry environment of Mornag_16 (Figure 1). In all three environments, lines derived from variety Imen were among the high yielders with a mean GY value of 2.69 t/ha outperforming significantly the lines acquired from Rihane, Manel, and Djebali by 26, 29, and 32%, respectively (Figure 1). Only lines from Ardhaoui showed similar high GY values to Imen derived lines, even though in one of the environments, El Kef_16, these lines indicated also a significantly lower GY value by 31% (Figure 1). The same pattern for GY was also depicted for the HI trait, for which lines originated from Imen showed a mean value of 0.43 across the three environments, being significantly higher from the mean HI values of Manel and Djebali derived lines by 19 and 30%, respectively (Figure 1). Concerning PH, lines originated from Djebali and Rihane were those that demonstrated the tallest stands with the differences being more profound in the Mornag_17 environment, where these lines showed mean PH values of 86.86 and 85.47 cm, respectively, surpassing the lines derived from Imen and Manel (Figure 1). Three distinct groups based on the source of the derived lines were shaped for SL. Lines originated from Manel and Djebali recorded the longest spikes with 7.49 and 7.44 cm, respectively, significantly higher from the group of Ardhaoui and Imen lines with 7.09 and 7.03 cm, as well as from the lines derived from variety Imen, which showed the shortest spikes with a mean value of 6.29 cm across the two environments that the measurement recorded (Figure 1). For the SGW, differentiation was found only in Mornag_17, where the lines acquired from Manel recorded a mean SGW value of 2.47 g being higher by 17, 19, and 22% from the respective values of the lines originated from Djebali, Rihane, and Ardhaoui (Figure 1). For PM, the lines acquired from Rihane showed higher susceptibility in the environment Kef_16 compared to all other lines recording average symptoms higher than the value of 3 in the disease scale (Figure 1). No differentiation was revealed for the TKW based on the source of the variety/landrace of the derived lines (Figure 1).

Among all agronomic traits, a significant genotype × environment interaction (G × E) was observed for GY, HI, and SGW. No significant G × E effects were detected for BY, PH, SL, TKW, and PM with the lines demonstrating a consistent performance across all the environments for these traits (Table 3). To a large extent, the significant G × E effects for GY were due to the contrasting performance of first-year LY lines, which were found to be the less productive lines in El Kef_16 and Mornag_16 environments showing a significant gap in GY compared to the first- and second-year HY lines by 47 and 41%, respectively, while ranked at the top as an average GY performance in the environment of Mornag_17, even though the differences with the other lines did not reach significance levels (Figure 1). An increase in HI at Mornag_17 environment was also apparent in the first year LY lines, since this index increased for these lines from 0.28 in El Kef_16 and Mornag_16 to 0.38 in Mornag_17, while all other lines maintained the same value of HI across all environments (Figure 1).

**FIGURE 2:**
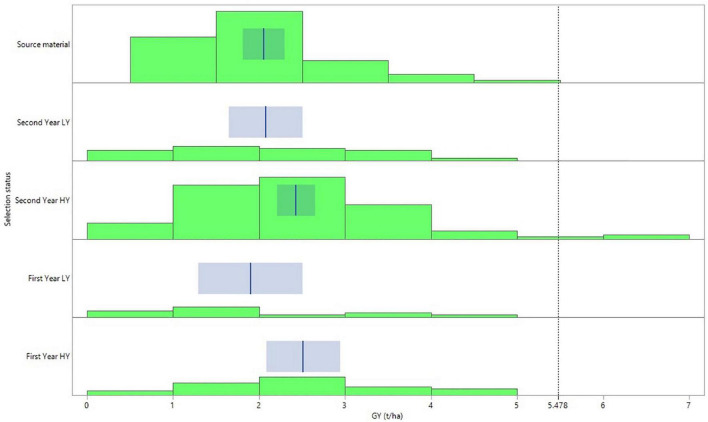
Distribution graph and fit of means for grain yield performance according to the selection status of the barley lines evaluated under dense stand trials in three environments in Tunisia.

Despite the significant G × E effect for GY, the general pattern across the three environments reflected with high consistency the selection status of the lines (Figure 2). Thus, the first- and second-year HY lines demonstrated the higher mean values for grain yield with 2.51 and 2.43 t/ha, significantly outperforming the source materials. The source materials in turn revealed the same GY mean value with the second year LY lines reaching at 2.05 and 2.07 t/ha, respectively. The least performing lines in terms of grain yield were the first-year LY lines with a mean GY of 1.90 t/ha across the three environments (Figure 2). Furthermore, the group of the second year HY lines was the only one in which some of the lines demonstrated a mean GY across the three environments that exceeded the right cutting-edge threshold value of the curve (5.478 t/ha), defined by the overall mean GY value plus three standard deviations (Figure 2).

**FIGURE 3:**
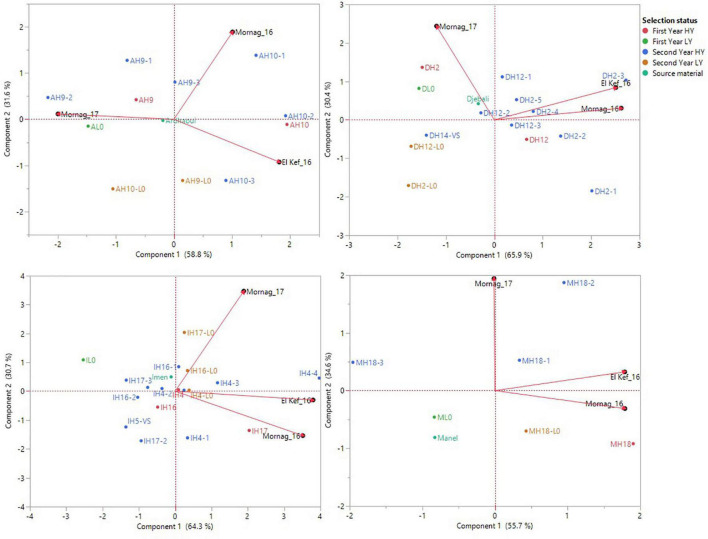
Source material-based GGE biplot analysis for grain yield performance of the barley lines evaluated under dense stand trials in three environments in Tunisia. Upper left: landraces Ardhaoui and derived lines, upper right: landrace Djebali and derived lines, bottom left: variety Imen and derived lines, bottom right: variety Manel and derived lines. Due to the limited number of lines derived from variety Rihane, GGE biplot analysis was not performed.

To avoid biased assumptions from a joint analysis due to the significant effects of the source material of the derived lines, GGE biplot analysis was performed separately for each of the different source varieties/landraces and their respective derived lines (Figure 3). Based on the analysis, the three environments were very contrasting for the lines derived from Ardhaoui and none of the lines recorded high grain yield in all three environments. Combining environments by two showed that most of the first- and second-year HY lines demonstrated high grain yield, while the first- and second- year LY lines, as well as the original population of Ardhaoui, either performed well only in one environment each time or their performance was poor for all the three environments (Figure 3). Four second-year HY lines from Djebali (DH2-3, DH2-4, DH2-5, and DH12-1) revealed high grain yield across all environments compared to their original population, with their scores to be plotted among the vectors that defined the three evaluation environments (Figure 3). Contrary, the second-year LY lines (DH2-L0 and DH12-L0) were the ones with the lowest grain yield among all Djebali lines (Figure 3). A more diverse pattern of selection status revealed high grain yield across the three environments for the lines derived from the variety Imen. Two second-year HY lines (IH4-4 and IH4-3) were predominantly the ones showing the highest grain yield for all three environments. Furthermore, line IH4-4 was the one that revealed consistently the highest grain yield for the three environments amongst all the entries tested, ranking first in El Kef_16 and third in Mornag_16 and Mornag_17 with a mean grain yield of 4.44 t/ha (Figure 3). Another one second-year HY line (IH17-1) and one second-year LY line (IH4-L0) derived from variety Imen showed good performance for grain yield for all three environments, while surprisingly three second-year HY lines (IH16-2, IH17-2, and IH5-VS) and one first-year HY line (IH16) were those with poor performance in all three environments (Figure 3). Having Manel as source material, two second-year HY lines (MH18-2 and MH18-1) showed consistency in terms of high grain yield in all the three environments, contrary to one first-year LY line and the source material of variety Manel that demonstrated low grain yield in each environment that the evaluation took place (Figure 3).

### Physiological Parameters

Regarding the physiological parameters, combined ANOVA revealed significant environmental effects for SPAD and LCT. Mornag_16 environment-induced higher values for the barley lines in comparison to El Kef_16 (Tables 5, 6). However, no significant G × E effects were revealed for none of the recorded physiological parameters in the trials (Table 5).

**TABLE 5:** Genotypic and environmental effects and their interaction on the physiological parameters of barley lines selected under ultra-low density when evaluated under dense stand trials in different environments in Tunisia.

Source of variation	DF	F_v_/F_m_	F_v_/F_0_	DF	SPAD	LCT
Entry	54	17.7***	34.0***	54	15.2***	26.3***
Environment	2	1.2	1.5	1	2.3***	1.5*
Entry × Environment	108	6.5	6.0	54	19.7	12.1

*Significant at α = 0.05; ***Significant at α = 0.001.

**TABLE 6:** Physiological parameters means and confidence intervals of barley entries evaluated in different environments in Tunisia.

	El Kef_16			Mornag_16			Mornag_17		
Trait	Mean	Lower 95%	Upper 95%	Mean	Lower 95%	Upper 95%	Mean	Lower 95%	Upper 95%
F_v_/F_m_	0.66	0.651	0.663	0.69	0.684	0.702	0.70	0.689	0.711
F_v_/F_0_	1.97	1.921	2.028	2.36	2.268	2.455	2.41	2.302	2.515
SPAD	42.48	41.760	43.195	51.66	51.061	52.256	NA	NA	NA
LCT	20.11	19.380	20.839	23.77	23.473	24.073	NA	NA	NA

**FIGURE 4:**
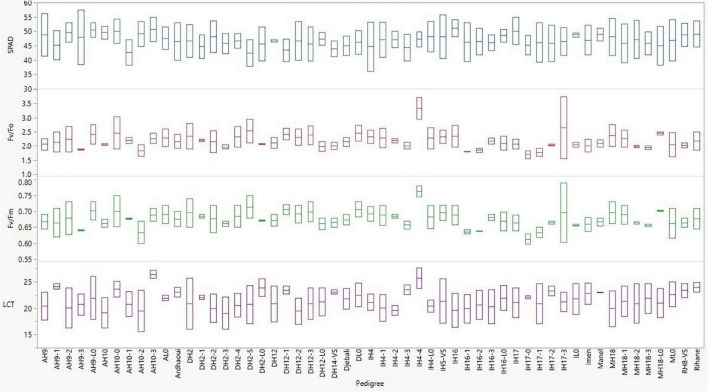
Entry box plots for the physiological parameters measured during the evaluation of the barley lines in the distinct environments in Tunisia.

Significant entry effects were revealed for these physiological traits (Table 5). Across all environments, the second year HY line IH4-4, which showed a consistent elite performance in terms of grain yield, was the one that exhibited the highest values for the ratios related to the photosynthetic activity with 0.76 for F_v_/F_m_ and 3.3 for F_v_/F_0_, significantly higher than the respective ratios of almost all other lines (Figure 4). Meanwhile, its source variety Imen was ranked among the entries that showed the lowest ratios for the two parameters of PSII (Figure 4). No other specific pattern, however, was observed, by means of selection status or source materials from which the lines were derived, regarding the F_v_/F_m_ and F_v_/F_0_ ratios (Figure 4). For LCT the second year HY line IH4-4 was again the one indicating the highest value among all other lines with a mean leaf canopy temperature of 25.7°C across all environments (Figure 4). Even though there was no specific pattern for LCT in terms of selection status or source material from which the lines were derived, a trend for high LCT values was observed for all the original varieties/landraces that were ranked among the top entries indicating high mean temperature values (Figure 4). As for the SPAD parameter, a trend for low SPAD values appeared for the lines derived by Djebali with a mean of 45.66. However, this trend did not reach a significant level when lines from Djebali were compared to the lines of other source materials (Figure 4).

### Grain Quality Parameters

Significant environmental effects on grain quality were detected for seed colour parameters as well as for the starch content (Table 7). The environment of Mornag_16 favoured the colour lightness and colour intensity of the grains with the three-colour parameters recording mean values of 152.55 for CLR_a, 119.47 for CLR_b, and 174.68 for CLR_L, significantly higher than the ones in El Kef_16, where the mean values for CLR_a, CLR_b, and CLR_L were 148.90, 114.05, and 172.29, respectively (Table 8). The starch grain content appeared to be higher in El Kef_16 with a mean value of 51.67% surpassing the respective mean starch content value of 50.43% in the Mornag_16 environment (Table 8). Regarding the G × E interaction, only a few of the considered grain quality parameters showed a significant effect. Thus, significant G × E interactions were limited to CLR_b and β-GLC, while all other grain quality parameters did not reveal any interaction between the barley lines and the environment (Table 7).

**TABLE 7:** Genotypic and environmental effects and their interaction on the grain quality parameters of barley lines selected under ultra-low density when evaluated under dense stand trials in different environments in Tunisia.

Source of variation	DF	PRM	LNG	WDT	CLR_a	CLR_b	CLR_L	PRT	STRCH	β-GLC
Entry	54	15.8***	16.5***	5.5***	8.4***	7.6***	7.6***	12.1***	11.4***	16.5***
Environment	1	28.5	31.6	2.7	3.2*	2.8**	2.8**	2.2	2.7*	30.8
Entry × Environment	54	28.4	31.4	13.9	14.5	15.1*	12.8	20.0	15.9	30.6*

*Significant at α = 0.05; **Significant at α = 0.01; ***Significant at α = 0.001.

**TABLE 8:** Grain quality parameters means and confidence intervals of barley entries evaluated in different environments in Tunisia.

	El Kef_16			Mornag_16		
Trait	Mean	Lower 95%	Upper 95%	Mean	Lower 95%	Upper 95%
PRM (mm)	31.05	30.545	31.555	30.59	30.133	31.041
LNG (mm)	10.50	10.314	10.684	10.31	10.145	10.473
WDT (mm)	2.92	2.902	2.949	2.95	2.929	2.977
CLR_a	148.90	148.231	149.559	152.45	151.577	153.318
CLR_b	114.05	113.065	115.035	119.57	118.516	120.620
CLR_L	172.29	171.707	172.880	174.68	173.952	175.404
PRT (%)	10.47	10.279	10.665	10.59	10.378	10.806
STRCH (%)	51.67	51.371	51.964	50.43	50.200	50.669
β-GLC (%)	4.46	4.147	4.768	4.63	4.328	4.922

**FIGURE 5:**
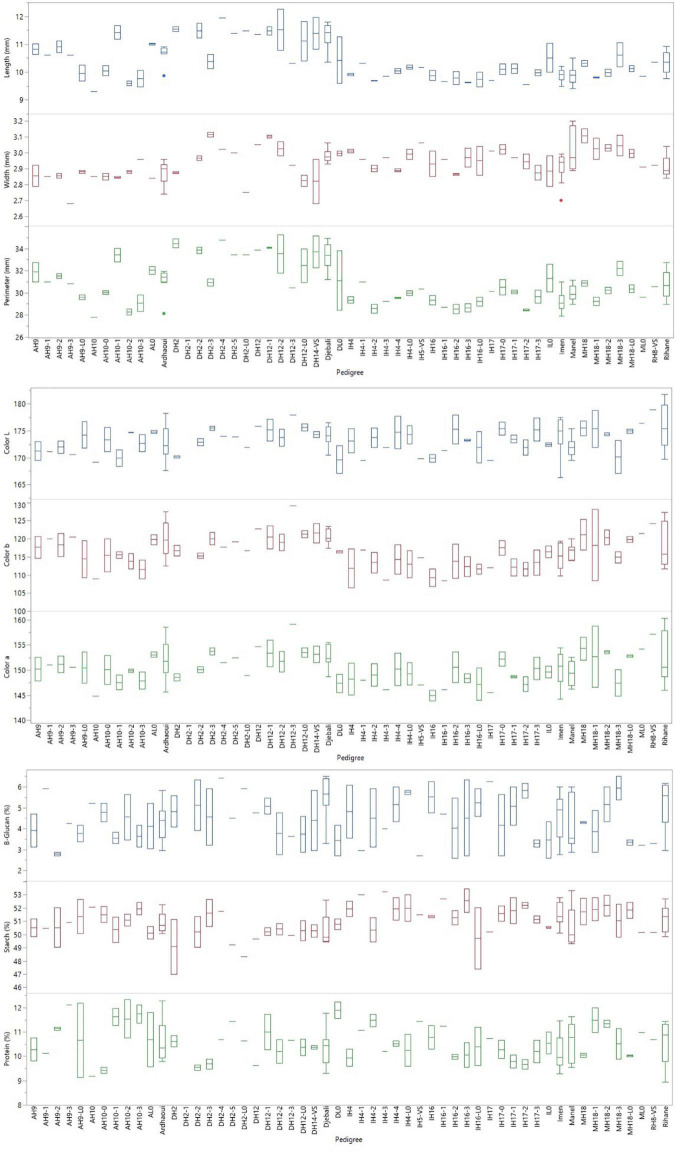
Entry box plots for the grain quality parameters measured during the evaluation of the barley lines in the distinct environments in Tunisia.

Highly significant entry effects were indicated for all the grain quality parameters, from the grain shape and size (PRM, LNG, WDT) up to the colouration (CLR_a, CLR_b, CLR_L) and seed nutrient content (PRT, STRCH, β-GLC) (Table 7). A clear trend based on the source materials that the lines derived was observed for the grain shape and size traits. Lines originating from Djebali showed significantly longer grains than all other lines with a mean LNG value of 11.24 mm. On the contrary, lines derived from variety Imen were the ones with the shortest grain length with a mean value of 9.93 mm (Figure 5). Djebali lines also showed a high value for grain width ranked second after the lines acquired from Manel for the specific trait. Thus, the mean WDT values for lines acquired from Manel was 3.02 mm, significantly higher than the value of 2.97, which was the mean value of lines derived from Djebali (Figure 5). The high LNG and WDT values from Djebali lines had a direct impact on the grain perimeter for which these lines were top-ranked with a mean value of 33.14 mm with a difference of a minimum of 3 mm in terms of perimeter compared to all other lines (Figure 5).

Regarding the lightness of the grain colour, no specific trend was indicated for barley lines. Some of the lines originated from Manel and Djebali, such as DH12-3, DH2-3, DH12-L0, MH18, MH18-2 appeared to be the ones with the lighter grain colour indicating significantly higher values for CLR_L compared to most of the other lines (Figure 5). However, these differences were more profound and source material specific for colour intensity. In this case, lines originated from Djebali showed, on average, higher values for CLR_a and CLR_b with a mean of 152.13 and 119.75, respectively, while lines derived from Imen were the ones that had lower values among all entries with CLR_a mean value 148.99 and CLR_b mean value 113.54 (Figure 5).

An increase in grain protein content by 2.34% on average was also revealed for the second-year HY lines in comparison to their respective source materials (Figure 5). This increase was consistent among all different source varieties/landraces and was more profound in the case of Ardhaoui, for which the second year HY lines significantly surpassed the source material of Ardhaoui by 7.34% for grain protein content (Figure 5). At the same time, β-glucan content appeared to be reduced among second-year HY lines by 7.24% in comparison to their respective source materials (Figure 5). The trend was specific to the source material, since the second year HY lines derived from Ardhaoui showed significantly lower β-glucan content by 9.95% from their source material, while on the other side the second year HY lines from Manel found by 17.2% on average higher than original variety Manel in terms of β-glucan content (Figure 5). Regarding grain starch content, selected HY lines did not reveal, as a general trend, any difference from the source material. However, among all lines, some second-year HY lines derived from Imen (IH4-3, IH16-3, IH4-1, IH17-2, IH16-1, IH4-4) were identified to show significantly higher starch content values among tested entries (Figure 5). On the opposite side, the lines derived from Djebali independently their selection status, along with their original population were those with the lower values for grain starch content (Figure 5).

### Correlations Among Traits

**FIGURE 6:**
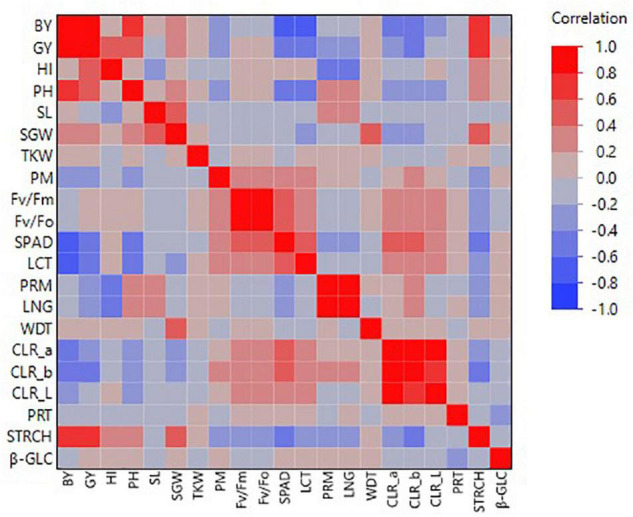
Colour map on pairwise correlations for the different traits recorded during the evaluation of the barley lines in the distinct environments in Tunisia.

Based on barley lines’ general performance, some distinct clusters of intercorrelated traits were revealed (Figure 6). In particular, GY was positively correlated with the agronomic performance traits of BY (*r* = 0.88), HI (*r* = 0.57), and PH (*r* = 0.47) (Figure 6). Surprisingly, no significant correlation was revealed between GY and TKW, as well as between GY and SL (Figure 6). Regarding the correlation to the physiological traits, GY was negatively correlated to SPAD (*r* = −0.42) and LCT (*r* = −0.46), even though for LCT the line that revealed consistently the higher grain yield across all environments was the one revealing the higher leaf canopy temperature (Figures 4, 6). On the other hand, the PSII related physiological traits, i.e., F_v_/F_m_ and F_v_/F_0_ did not show any correlation with GY (Figure 6). As far as the grain quality parameters, a significant correlation was found between GY and STRCH (*r* = 0.68), while significant negative correlations were found between GY and CLR_b colour intensity (*r* = −0.50) (Figure 6). Another interesting cluster for intercorrelated traits was the one shaped among the four measured physiological parameters (F_v_/F_m_, F_v_/F_0_, SPAD, LCT) for which the paired correlations were in all cases significant ranging from *r* = 0.41 (between SPAD and F_v_/F_m_) up to *r* = 0.98 (between F_v_/F_m_ and F_v_/F_0_) (Figure 6).

## Discussion

In this study, 50 barley lines selected by applying divergent single plant selection at ultra-low density within three commercial cultivars and two Tunisian landraces were evaluated in comparison to their source material in an open field under highly contrasting environmental conditions in Tunisia, ranging from dry (Mornag_16; 295 mm annual rainfall) to moderately dry (El Kef_16; 325 mm annual rainfall) up to favourable ones (Mornag_17; 458 mm annual rainfall). The results of this study revealed that the selection applied under ultra-low density reflected with high consistency the grain yield patterns under dense stands with the first- and second-year HY lines to outperform the source material, and the first year LY lines to rank under all entries in terms of grain yield. These lines were derived after applying intra-cultivar selection within source materials of different genetic backgrounds regarding their genetic constitution. That is, while some genetic diversity was expected to be exploitable within the two landraces, no genetic variation was expected theoretically to occur within the improved barley varieties. However, the present study revealed that even within improved varieties, individual plant selection under ultra-low density was efficient to select for HY lines that outperformed their respective source material across all environments.

Although intra-cultivar variation has long been recognised in crop species (Sprague et al., 1960; Russell et al., 1963; Byth and Weber, 1968), it is oftentimes ignored due to the common belief that elite cultivars are highly homogeneous (Fasoula and Boerma, 2007; Haun et al., 2011). Nevertheless, even within homogeneous gene pools, an intrinsic amount of latent genetic variation may still occur, whereas mechanisms that generate *de novo* variation may also be present. Residual heterozygosity, due to segregation of parental loci during the breeding process is presumably one source of genetic variation (Haun et al., 2011; Tokatlidis, 2015). On the other hand, additional heterogeneity might stem from *de novo* generated variation, resulting from spontaneous mutations (Shaw et al., 2000; Ossowski et al., 2010) or *via* genetic and epigenetic mechanisms, such as intragenic recombination, unequal crossing over, gene duplications, or deletions, DNA methylation, excision or insertion of transposable elements, chromatin alterations, and others (Rasmusson and Phillips, 1997; Sani et al., 2013; Cavrak et al., 2014; Kim and Zilberman, 2014).

Despite the wide variability in terms of annual precipitations among the three testing environments, no significant G × E interactions were found for most of the recorded traits. Thus, the barley lines selected under ultra-low density revealed a high buffering capacity for biological yield, demonstrating similar patterns for biomass production across all environments, regardless of the unpredictable precipitation rates. The plasticity of the selected lines as a response to environmental conditions was also maintained for other agronomic traits, such as plant height, spike length, thousand kernel weight, and powdery mildew resistance. However, a significant G × E effect was indicated for the grain yield mainly as a response to the strong G × E interactions for the harvest index and the grain weight per spike. Yet, as the high correlation to the biological yield entails the G × E effect for grain yield was marginally significant, implying a good buffering capacity of the selected lines for this particular trait, too. Furthermore, some selected lines were found to outperform their source material and the best checks across all environments consistently.

Buffering capacity is a crucial feature for the development of modern varieties, to tackle the unpredictable environmental conditions by making optimum use of available resources in both marginal and favourable environments. Defining the optimum planting density under these variable and fragile conditions to accomplish the attainable yield depends on many crop parameters, as well as on several factors related to the genotype itself and the applied cultivation practices. In maize, abiotic adversities show a more pronounced effect under dense stands (Berzsenyi and Tokatlidis, 2012; Solomon et al., 2017; Mylonas et al., 2020). On the other hand, Bastos et al. (2020), mentioned for wheat that under high-yielding and less limited resources environments the number of plants required to maximise yields was very low, below any commercial recommended number of plants for this crop. However, a higher planting density was needed for the low-yielding environments to sustain maximum yields (Bastos et al., 2020).

To this end, Tokatlidis et al. (2001) indicated the need to extend the lower and upper limits of optimum crop plant density. The authors highlight the concept of developing density-independent varieties that offer flexibility and plasticity to environmental diversity and secure over-season stability (Tokatlidis et al., 2001; Tokatlidis, 2017). Lower and upper limits of the optimum density are determined by individual plant yield efficiency and tolerance to high densities, respectively (Tokatlidis et al., 2001). Extending the lower limits of the optimum crop density has been proven more challenging than expected. Hence, evidence from research on maize suggests that plant yield potential of maize hybrids remained practically without significant change along the years of maize hybrid development, and it is the hybrid performance as a response to a steadily increasing density rate that is improved (Tollenaar and Lee, 2002; Tokatlidis and Koutroubas, 2004; Duvick, 2005; Gonzalez et al., 2018) or in the best-case scenario, a positive impact on yield components as other sources for yield gain was also identified (Assefa et al., 2018). Given the inverse relationship between the yield of a genotype and its competitive ability (Sedgley, 1991; Fasoula and Fasoula, 1997; Pan et al., 2003; Chatzoglou and Tokatlidis, 2012; Ninou et al., 2014), Tokatlidis (2017) introduced the idea of exploiting in plant breeding the “weak competitor” ideotype. In other words, since under dense stand conditions, the superiority of a plant that stands out could stem from being a strong competitor, while a weak neighbour devoid of genetic competitive ability might be the one with the higher yield potential, Tokatlidis (2017) recommended evaluation and selection of individual plants adequately spaced under a regime that simulates conditions of nil-competition. Evaluation of genotypes under ultra-low density in a regime that practically resembles nil-competition has been successfully also applied as a predictive tool for plant yield efficiency and stability (Sinapidou et al., 2020). Our findings confirm the above remarks, since selection under ultra-low density for high plant yield efficiency, resulted in the selection of superior barley lines with enhanced buffering capacity, revealing high stability in unpredictable environments that ranged from dry (Mornag_2016) up to favourable (Mornag_2017) ones.

Correlation between physiological parameters and agronomic performance traits for the evaluated barley lines showed variable results. According to Fang and Xiong (2015), to overcome drought stress at the physiological level, plants adjust their rates of photosynthesis by modifying photosystem II, inducing the stomatal closure, and lowering the carbohydrate and nitrogen metabolism, as well as the nucleic acid, and protein activity. The effect of drought stress on PSII in plants has been found controversial. Hence, while in some studies chlorophyll fluorescence was found to be useful to evaluate yield performance under rainfed Mediterranean conditions in durum wheat (Araus et al., 1998) and barley (Li et al., 2006), in some others it has been considered as of limited use (Aberkane et al., 2021). On the other hand, leaf canopy temperature has been reported as a useful criterion to select for water-stressed environments and a high correlation has been found between lower canopy temperature and grain yield in wheat (Amani et al., 1996; Reynolds et al., 1998). A significant negative correlation between grain yield and leaf canopy temperature was also revealed from our study, implying that higher grain yield was associated with lower canopy temperature. However, it is worth mentioning that the line which outperformed consistently all other lines across all environments was the one that showed the higher leaf canopy temperature among all the evaluated entries, meaning that other factors are also crucial to determine efficient response to drought conditions. Regarding chlorophyll content, a significant negative correlation was observed between grain yield and SPAD values, which was not expected based on some research evidence that drought and heat stress affect the photosynthetic activity by reducing chlorophyll content (Feng et al., 2014; Sangwan et al., 2018). However, other researchers have reported limited or no association between chlorophyll content and grain yield under heat and drought stress conditions (Pinto et al., 2010; Aberkane et al., 2021).

Good plasticity of barley lines was also indicated for the grain quality parameters since no G × E effects were revealed for most of the recorded quality traits. Furthermore, the improved agronomic performance of the barley selected lines, induced an indirect positive effect on grain protein content with most of the selected high yielding lines to maintain or even improve their protein content in comparison to their source material. Such results are very promising, particularly under the view of a global trend that has been reported toward the lowering of grain quality in high yielding agronomic conditions and among modern cultivars, because breeders are selecting for grain yield but not for quality (Fan et al., 2008; Laidig et al., 2017; Marcos-Barbero et al., 2021). Nevertheless, as Simmonds (1996) highlighted, despite the consensus for strongly negative correlations between grain yield and protein content in cereals a positive expected relationship also holds by making, however, some compromises between attainable high yield or high protein content. The results of our study indicated that small progress in grain protein content has been achieved, while selecting for high grain yield, in accordance with Simmonds’s (1996) remark. Working with lentil crops, following a 2-year selection cycle for individual plant yield under ultra-low density, Ninou et al. (2019) ensured that the selection of high yielding lines maintained or even improved their seed quality characteristics.

Overall, the development of varieties with enhanced buffering capacity, characterised by density independence and resource use efficiency is of utmost importance for the farmers to sustain the yield under the unpredictability and inter-annual variation of agricultural environments. Toward this direction, selection for plant yield efficiency at ultra-low-density conditions sounds like a prudent tool to narrow the gap between the actual and the attainable yield and to meet future challenges in agriculture.

## Conclusion

Considering the challenges imposed by climate variation and volatility of agricultural environments, the development of modern cultivars with high and stable performance across a wide range of environments is an imperative need. The results of our study revealed that selection for high plant yield efficiency under ultra-low density resulted in the development of high yielding lines with an innate buffering capacity, outperforming their source materials and the best checks consistently under contrasting environments. In addition, the potential at the nil-competition regime for efficient selection within narrow gene pools has been well demonstrated. Furthermore, results suggest that single-plant selection under ultra-low density could serve as an effective strategy for developing high-yielding barley varieties maintaining concurrently a high grain quality profile.

## Data Availability Statement

The raw data supporting the conclusions of this article will be made available by the authors, without undue reservation.

## Author Contributions

AT drafted the manuscript. AT and HBG conceptualised the study and designed the field trials. HBG coordinated the field trials and the collection of agronomic and physiological traits. AE-B performed the grain quality analysis and collected and curated the quality data. ZK and AT coordinated data curation and performed the statistical analysis. All authors contributed to the development of the entire manuscript and reviewed and edited the final version.

## Conflict of Interest

The authors declare that the research was conducted in the absence of any commercial or financial relationships that could be construed as a potential conflict of interest. The reviewer IM declared a past co-authorship with one of the authors AT to the handling editor.

## Publisher’s Note

All claims expressed in this article are solely those of the authors and do not necessarily represent those of their affiliated organizations, or those of the publisher, the editors and the reviewers. Any product that may be evaluated in this article, or claim that may be made by its manufacturer, is not guaranteed or endorsed by the publisher.
